# Cyclohexane extract of walnut leaves improves indices of oxidative stress, total homocysteine and lipids profiles in streptozotocin‐induced diabetic rats

**DOI:** 10.14814/phy2.14348

**Published:** 2020-01-21

**Authors:** Gholamali Jelodar, Masoud Mohammadi, Abolfazl Akbari, Saeed Nazifi

**Affiliations:** ^1^ Department of Physiology School of Veterinary Medicine Shiraz University Shiraz Iran; ^2^ Department of Clinical Studies School of Veterinary Medicine Shiraz University Shiraz Iran

**Keywords:** cyclohexane extract, diabetes mellitus, lipid profile, oxidative stress, walnut leaf

## Abstract

This study aimed to evaluate the effect of two doses of cyclohexane extract of walnut leaves on total homocysteine, lipids profiles, and indices of oxidative stress including superoxide dismutase (SOD), glutathione peroxidase (GPx), catalase (CAT), and malondialdehyde (MDA) in diabetic rats. Diabetes was induced by a single intraperitoneal (IP) injection of streptozotocin (50 mg/kg BW). Twenty‐eight male Sprague Dawley rats were randomly divided into four groups, group I: control (received sesame oil as vehicle), group II: diabetic control (received sesame oil), group III and IV: diabetic rats treated by 150 and 250 mg/kg body weight (BW) per day extract of walnut leaves, respectively. All groups were treated for 28 days via oral gavage. Fasting blood glucose (FBG) level and body weight measured before injection, 3 days after injection, and on days 0, 7, 14, 21, and 28 of treatment. At the end the 28th day of the experiment, blood samples collected via heart puncture and the sera were used for estimation of the above‐mentioned parameters.** **The results showed a decrease in FBS, TC, TG, LDL‐c, VLDL‐c, homocysteine, and MDA level and increase in the level of HDL‐c in diabetics treated by walnut leave extracts in a dose‐dependent manner after 28 days. The activity of antioxidant enzymes significantly increased in treated groups compared with diabetic control. It can be concluded that cyclohexane extract of walnut leaves has an overall beneficial effect on body weight, fasting blood glucose, lipids profile, antioxidant enzyme activities, and homocysteine.

## Graphical abstract

Two doses of cyclohexane extract of walnut leaves on total homocysteine, lipids profiles and indices of oxidative stress were evaluated. Beneficial effects on body weight, fasting blood glucose, lipids profile, antioxidant enzyme activities, and homocysteine were observed.

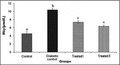

## 1 INTRODUCTION

Diabetes mellitus (DM) is a heterogeneous endocrine and metabolic disease characterized by hyperglycemia frequently accompanied by glycosuria, polydipsia, and polyuria and other metabolic disorder (American Diabetes Association, 2009). This disease develops as a result of the deficiencies in the insulin level or function or both and usually is related to specific changes in intracellular metabolism and morphological changes in the kidney, retina, pancreas, and other organs (American Diabetes Association, 2009; Cantley & Ashcroft, 2015). The incidence of Diabetes mellitus is rapidly increasing and has become a major public health problem. Currently, about 4.4 million Iranian people have fasting hyperglycemia and two million of them are suffering from DM complications (Rashidi, Mirhashemi, Taghizadeh, & Sarkhail, 2013). Many factors are involved in the pathogenesis of diabetes mellitus and its complications (Lipinski, 2001; Yang, Jin, Lam, Wai, & Yan, 2011) and one of the most important is oxidative stress. Oxidative stress refers to the excessive generation of free radicals and depletion of free radical scavenging enzymes that have been demonstrated in animals and in human subjects with diabetes mellitus (Matough, Budin, Hamid, Alwahaibi, & Mohamed, 2012). Formation of reactive oxygen species (ROS) leads to β‐cell dysfunction initiated by inflammatory cytokines and autoimmune reactions in type 1 of DM (Cernea & Dobreanu, 2013; Matough et al., 2012). In type 2 DM, β cell apoptotic pathways, impair insulin synthesis and also insulin resistance is activated by ROS formation (Cernea & Dobreanu, 2013). Therefore, this enhances the body's antioxidant system by the use of supplements and plant compounds can improve the status of oxidative stress and prevent the onset of these diseases. Although oral hypoglycemic substances are effective for blood glucose control at least in the early stages of diabetes, they may not be effective in preventing the progression of organ damage mediated by ROS (Rojas & Gomes, 2013). Another mechanism that may have a role in diabetes complications is hyperhomocysteinemia. Homocysteine (Hcy) is a nonprotein‐forming sulfur amino acid that originated from the metabolism of methionine. Hcy metabolism is mostly controlled by epigenetic regulation such as DNA methylation, histone modifications, and acetylation (Kamat, Mallonee, George, Tyagi, & Tyagi, 2016). The elevated level of plasma Hcy has been identified as an atherogenic agent, promoting endothelial dysfunction, cell proliferation, oxidative stress, inflammation, and thrombosis (Pushpakumar, Kundu, & Sen, 2014). Elevated homocysteine via interference metabolic pathways impairs physiological mechanisms in the body. Besides, hyperhomocysteinemia can induce the production of ROS and lead to oxidative damage.

Today it is well demonstrated that the use of herbal supplements due to phenolic and flavonoids compounds can play a significant role in the prevention and treatment of many metabolic diseases. Walnut leaf (*Juglans regia L.*) contains phytochemical compounds that have strong antioxidant (Jelodar, Mohsen, & Shahram, 2007), anti‐inflammatory (Hosseinzadeh, Zarei, & Taghiabadi, 2011), antidiabetic, hypolipidemic (Delaviz, Mohammadi, Ghalamfarsa, Mohammadi, & Farhadi, 2017; Hosseini et al., 2014), and anti‐carcinogenic activity (Shah, Sharma, & Shah, 2015). Many studies have shown that the use of walnut leaf in the form of aqueous‐alcoholic extract, alcoholic, cyclohexane, and powder decrease blood glucose level in animal and human with diabetes mellitus (Delaviz et al., 2017; Hosseini et al., 2014; Jelodar et al., 2007). It was reported that dietary intake of cyclohexane, ether, and ethanol extracts of walnut leaves decreases the concentration of glucose, cholesterol, triglyceride, and serum urea nitrogen (Hosseini et al., 2014; Jelodar et al., 2007). Polar (ethanol) and nonpolar (cyclohexane) solvents may release different effective materials found in the walnut leaf.

Although many studies have shown the prevention and therapeutic role of walnut leaf extract on diabetes mellitus, few studies have been done on the therapeutic role of cyclohexane extract of the walnut leaf on total homocysteine in streptozotocin (STZ)‐induced diabetic rat. Hence, this study was designed to investigate the effect of oral administration of different doses of cyclohexane extract of the walnut leaf on body weight, blood glucose, lipid profiles, and total homocysteine in normal and diabetic rats.

## 2 MATERIALS AND METHODS

### 2.1 Plant material and extraction

Walnut leaves were collected during the month of June in Fars province from a farm of Agricultural College of Shiraz University Iran. The walnut leaves were dried in the shade (22 ± 2°C) for about 72 hr. Dried leaves were ground into a fine powder using a homogenizer. The cyclohexane extract of walnut leaves was determined according to our previous report (Jelodar et al., 2007).

### 2.2 Animals

Twenty‐eight male Sprague Dawley rats, weighing approximately 250 ± 20 g were used. Animals were kept in polypropylene cages under standard conditions: temperature (22 ± 2°C), relative humidity (38%), and 12/12 hr light/dark cycle, and had free access to standard pellets diet and water.

### 2.3 Animal ethics

All aspects of this study were approved by the state committee on animal ethics, Shiraz University, Shiraz, Iran. Also, the recommendations of the European Council Directive (86/609/EC) of November 24, 1986, regarding the standards in the protection of animals used for experimental purposes were followed.

### 2.4 Induction of diabetes and experimental design

In order to induce DM in rats, the animals fasted overnight and a single intraperitoneal injection of a freshly prepared solution of streptozotocin (STZ; 50 mg/kg BW) in 0.1 M citrate buffer (pH = 4.5) was used. A glucose solution (%5) was prepared for animals overnight to prevent the drug‐induced hypoglycemia; control rats received citrate buffer injection alone. The animals with blood glucose >250 mg/dl on the 3rd day after STZ injection were considered diabetic. The treatment was started on the 3rd day after STZ injection and this was considered as the 1st day of treatment. The selected dose of walnut leaf extract was according to our previous report (Jelodar & Nazifi, 2001). The rats were allocated into four groups comprising seven animals in each group as follows:

Group I: Control rats received 0.1 M citrate buffer (pH= 4.5) only at the start of the study and then sesame oil orally by gavage.
Group II: Diabetic controls; received sesame oil orally by gavage.
Group III: Diabetic treatment I; received walnut leaves (150 mg/kg of BW/day) orally by gavage.
Group IV: Diabetic treatment II; received walnut leaves (250 mg/kg of BW /day) orally by gavage.

The treatment was continued for 28 days, and blood glucose and body weight were evaluated in days of 0, 7, 14, 21, and 28 of the study period.

#### 2.4.1 Serum and hemoglobin preparation for lipid profiles, glucose, Hcy, MDA detection, and enzymes assay

On the last day of the experiment, the rats were anesthetized using diethyl ether. Blood samples were collected via heart puncture. Serum was used for the estimation of glucose, cholesterol, HDL‐c, LDL‐c, VLDL‐c, and TG, and Hcy concentrations. In order to remove plasma components, the heparinized blood was centrifuged (at 2000 *g* for 5 min, Centrifuge 5415 R; Rotofix 32A, Germany). The packed red cells were washed three times in an isotonic saline solution (0.9% NaCl) and red cells were osmotically lysed with 2 ml cold distilled water and used for antioxidant enzyme activity and MDA. Hemoglobin (Hb) was measured using the cyanmethemoglobin method.

#### 2.4.2 Measurement of blood glucose and serum lipids

Blood glucose level was measured by an automated digital glucometer (Accu‐Chek Advantage). Lipid profiles including, total cholesterol (TC) and total triglycerides (TG), low‐density lipoprotein (LDL‐c), and high‐density lipoprotein (HDL‐c) were evaluated according to the manufacturer's instructions using kits supplied by Pars Azmun Co.

### 2.5 Measurement of total homocysteine (tHcy) concentration

Total homocysteine of serum, which refers to the sum of protein‐bound, free‐oxidized, and reduced species of homocysteine, was determined by the Axis® Homocysteine ELISA kit. The wavelength of 450 nm was used to evaluate tHcy by ELISA reader (STAT FAX 2100, USA). All estimations were performed in duplicate and the intra‐assay coefficient of variation was <10%.

### 2.6 Measurement of superoxide dismutase (SOD) and glutathione peroxidase (GPx) activity

SOD and GPx activities were evaluated with SOD and GPx detection kit (Ransod kit and Ransel kit for SOD and GPx, respectively, produced by Randox Co. UK) according to the manufacturer's instructions. The SOD or GPx activity was expressed as unit per mg of Hb (U/g Hb).

### 2.7 Measurement of catalase (CAT) activity

Catalase activity was assayed spectrophotometrically by monitoring the decomposition of H_2_O_2_ using the procedure of Aebi (Aebi, 1984) and applied in our previous study (Jelodar, Akbari, & Nazifi, 2013).

### 2.8 Measurement of lipid peroxidation (MDA)

To evaluate lipid peroxidation in blood, the malondialdehyde level was evaluated by a modified HPLC method which is based on the reaction of MDA with thiobarbituric acid to form a colored MDA‐TBA adduct as previously described (Jelodar et al., 2013; Lykkesfeldt, 2001).

### 2.9 Statistical analysis

The results are presented as means ± standard error of the mean (Mean ± *SEM*). All data were recorded with the Statistical Package for Social Sciences (SPSS‐19.0). One‐way analysis of variance (ANOVA) was used to analyze the data, followed by post hoc multiple comparisons Tukey test for comparison between different treatment groups. Statistical significance was set at *p* < .05.

## 3 RESULTS

**Figure 1:**
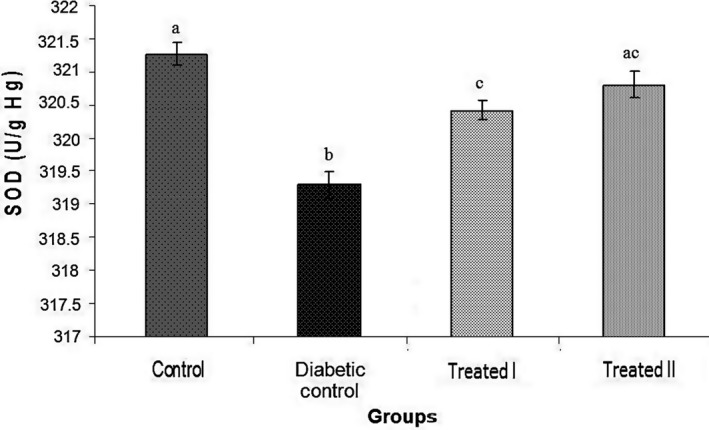
Treatment of diabetic rats by walnut leaf extracts significantly increased the activity of superoxide dismutase (SOD) activity. Values represent mean ± *SEM* in seven replications. Different alphabets show a significant difference with other groups (*p* < .05). Groups with the similar alphabet on each column are not significantly different, but different alphabets indicate a significant difference between groups, the label of “ac” means that it has no significant difference with columns labeled as “a” and “c

**Figure 2:**
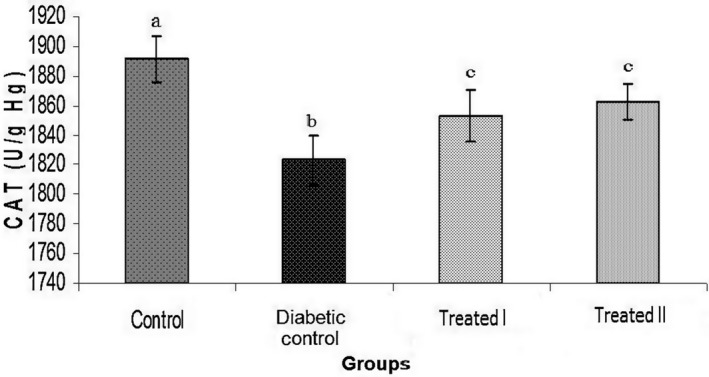
Comparison of catalase (CAT) activity among different groups. Values represent the mean ± *SEM* in seven replications. Different alphabets show a significant difference with other groups (*p* < .05). Groups with a similar alphabet on each column are not significantly different, but different alphabets indicate a significant difference between groups

**Figure 3:**
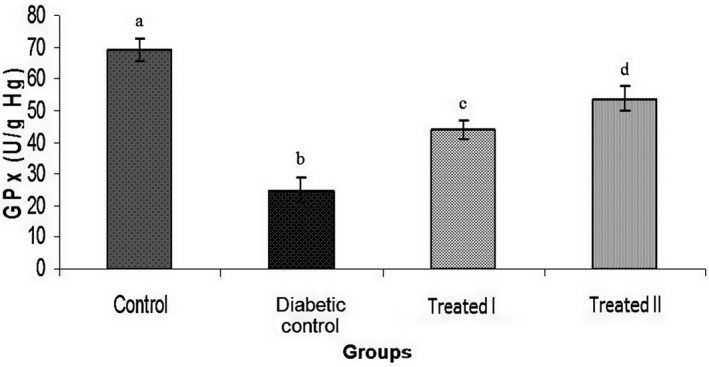
Glutathione peroxidase (GPx) activity increases significantly in diabetic rats following treatment by walnut leaf extracts. Values represent the mean ± *SEM* in seven replications. Different alphabet shows a significant difference with other groups (*p* < .05). Groups with a similar alphabet on each column are not significantly different, but different alphabets indicate a significant difference between groups

**Figure 4:**
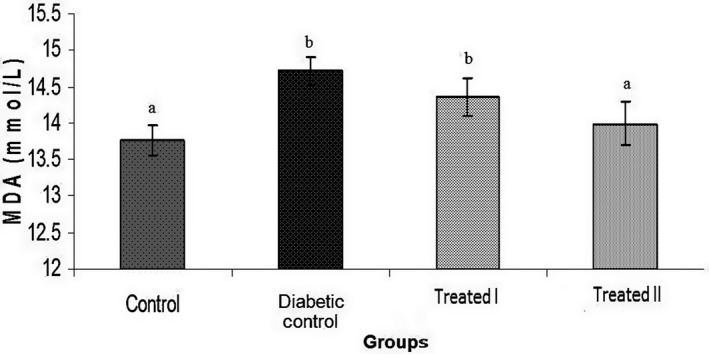
Comparison of malondialdehyde (MDA) activity among different groups. Values represent mean ± *SEM* in seven replications. Different alphabet shows a significant difference with other groups (*p* < .05). Groups with a similar alphabet on each column are not significantly different, but different alphabets indicate a significant difference between groups

**Figure 5:**
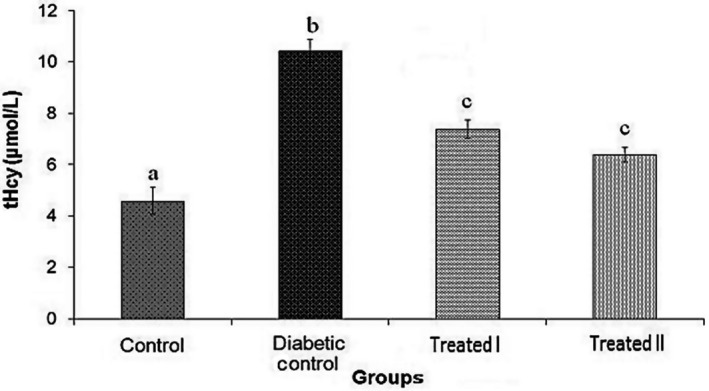
Total Homocysteine (tHcy) level decreased significantly in diabetic rats following 28 days of treatment by walnut leaf extracts. Values represent mean ± *SEM* in seven replications. Different alphabet shows a significant difference with other groups (*p* < .05). Groups with a similar alphabet on each column are not significantly different, but different alphabets indicate a significant difference between groups

In this study, doses of 150 and 250 mg/kg of the extract of walnut leaves were used. The results show that there were statistically significant differences in body weight between all groups at the end of the study (Table 1). FBG level was measured on days 0, 7, 14, and 28 days after confirming of induction of diabetes mellitus. The results of FBG on day 7 did not show an improvement in hyperglycemia (Table 2). However, administration of extract of walnut leaves improved FBG level in the treatment group I (150 mg/kg) and treatment group II (250 mg/kg) in a dose‐dependent manner (*p* < .05), and had a significant effect on BW of the rats at the end of the study (Tables 2). Treatment with extract of walnut leaves resulted in a significant decrease in cholesterol, TG, VLDL‐c and LDL‐c concentrations in a dose‐dependent manner (Table 3). In diabetic rats, a dose‐dependent increase in the level of HDL‐c was also observed (Table 3). The mean values (±*SEM*) of GPx, SOD, CAT activities and MDA (as the biomarker for lipid peroxidation) in the rat erythrocyte are presented in Figures 1, 2, 3, 4. In the diabetic rat, a significant decrease in the activity of these enzymes was seen in the diabetic‐control group compared to other groups, while the administration of extract of walnut leaves could significantly increase the activity of these enzymes and bring them to the normal level. The significant increase in concentration of MDA and homocysteine observed in the red blood cells of the diabetic rats reverted to near‐normal levels in the diabetic rats treated with the cyclohexane extract of walnut leaves. Levels of red blood cells MDA and homocysteine were reduced in a dose‐dependent manner at the end of the experimental period (Figures 4 and 5).

**Table 1:** The mean (±*SEM*) of body weight (g) changes in different groups during 28 days

Group	Day 0	Day 3	Day 7	Day 14	Day 21	Day 28
Control	262.67 ± 7.14aA	270.33 ± 7.24aAB	279.33 ± 7.28aB	296.67 ± 7.59aC	313.00 ± 7.79 aD	331.67 ± 7.61aD
Diab‐control	262.15 ± 3.16aA	256.5 ± 2.95aA	248.33 ± 3.37bB	232.83 ± 3.16bC	216.67 ± 3.11 bD	199.69 ± 2.99bE
Treated l	265.67 ± 4.60aA	259.17 ± 4.65aA	252.68 ± 4.67bA	256.33 ± 4.58cA	271.17 ± 6.80 cA	276.20 ± 6.74cA
Treated ll	265.17 ± 4.36aA	259.13 ± 4.15aA	254.33 ± 4.42bA	261.33 ± 4.2 cA	271.83 ± 4.21cAB	284.25 ± 3.90cB

Note

The results were analyzed using one‐way analysis of variance (ANOVA) followed by post hoc multiple comparisons Tukey test for comparison between different treatment groups Different capital alphabetic letters show significant differences among days of evaluation (*p* < .05). Groups with the similar alphabet on each column or row are not significantly different, but different alphabets indicate a significant difference between groups, the label of “AB” means that it has no significant difference with columns labeled as “A” and “B”.

**Table 2:** Changes in fasting blood glucose (mg/dl) concentrations in different groups during 28 days of the experimental period

Group	Day 0	Day 3	Day 7	Day 14	Day 21	Day 28
Control	105.17 ± 2.66aA	92.96 ± 1.99aA	96.51 ± 1.74aA	94.17 ± 1.71aA	99.17 ± 2.12aA	95.33 ± 2.17aA
Diab‐Control	101.83 ± 6.76aA	407.66 ± 5.60bB	432.83 ± 5.78cC	454.83 ± 4.48bCD	477.17 ± 3.97bD	531.51 ± 5.23bE
Treated l	96.50 ± 4.29aA	405.67 ± 4.49bB	404.33 ± 5.71bB	388.17 ± 3.97cC	377.67 ± 4.38cC	366.56 ± 5.23cC
Treated ll	99.33 ± 2.61aA	411.67 ± 2.94bB	395.01 ± 3.74cC	378.01 ± 3.65cD	353.33 ± 3.96cD	330.92 ± 5.02cE

Note

The results were analyzed using one‐way analysis of variance (ANOVA) followed by post hoc multiple comparisons Tukey test for comparison between different treatment groups. Different capital alphabetic letters show significant differences among days of evaluation (*p* < .05). Different small alphabetic letters show significant differences among groups.

**Table 3:** A 28‐day treatment using walnut leaf extracts significantly reduced serum lipid concentrations in diabetic rats in a dose‐dependent manner

Group	Cholesterol(mg/dl)	Triglycerides(mg/dl)	LDL (mg/dl)	HDLmg/dl)	VLDL (mg/dl)
Control	25.17 ± 1.13a	42.51 ± 0.26a	5.83 ± 0.26a	37.96 ± 1.31a	7.32 ± 0.53a
Diab‐Control	61.83 ± 5.68b	114.55 ± 2.89b	10.63 ± 0.27b	20.42 ± 1.77b	23.4 ± 0.39b
Treated l	42.42 ± 0.79c	91.44 ± 1.51c	8.71 ± 0.18c	24.13 ± 1.59b	17.62 ± 0.59c
Treated ll	32.84 ± 0.98ac	63.63 ± 3.58d	7.02 ± 0.25d	30.33 ± 0.59c	12.44 ± 0.64d

Note

The results were analyzed using one‐way analysis of variance (ANOVA) followed by post hoc multiple comparisons Tukey test for comparison between different treatment groups. Different alphabetic letters show significant differences among groups(*p* < .05). Groups with the similar alphabet on each column are not significantly different, but different alphabets indicate a significant difference between groups, the label of “ac” means that it has no significant difference with columns labeled as “a” and “c”.

## 4 DISCUSSION

In this study, the effect of two oral doses of cyclohexane extract of walnut leaves on body weight, glucose, serum lipid profile, homocysteine, and indices of oxidative stress in streptozotocin‐induced diabetic rats was evaluated. In the diabetic group, weight loss was significantly compared with nondiabetics, which is in agreement with other reports (Abbasi, Jelodar, & Nazifi, 2017; Rajkumar, Srinivasan, Balasubramanian, & Govindarajulu, 1991). Weight loss in diabetic rats may result from the destruction or decomposition of protein structure (Rajkumar et al., 1991). It is rational in the absence of insulin, cells, especially skeletal muscle cells cannot use glucose and consume intracellular proteins as energy sources. The importance of oxidative stress in the pathogenesis and DM complications is well studied and documented (Giacco & Brownlee, 2010; Matough et al., 2012). Increasing free radicals generation and high level of oxidative stress due to depletion of the activity of free radical scavenging enzymes caused by chronic hyperglycemia exhibited in both human and experimental animal models of diabetes (Giacco & Brownlee, 2010). Hyperglycemia leads to the production of free radicals which will induce oxidative stress. hyperglycemia may activate protein kinase C (PKC) through different mechanisms, including activation of phospholipase C, synthesis of diacylglycerol (DAG), and inhibition of DAG kinase (Koya & King, 1998). PKC increases oxidative damage by activating mitochondrial NADPH oxidase (Koya & King, 1998). Oxidative damage in the erythrocytes can lead to loss of cell function. Excessive lipid peroxidation damages the fluidity of cell membranes and alters the activity of the membrane's enzymes and receptors leading to membrane malfunction (Roy et al., 2016). The high level of MDA, as a biomarker for lipid peroxidation, in the diabetic control rats is a reflection of the insufficiency of antioxidant defenses in combating ROS‐mediated damage. Treatment of rats by administration of different doses of extract of walnut leaves causes an increase in the activity of glutathione peroxidase, superoxide dismutase and catalase in red blood cells and suppressed MDA concentration significantly. Our results also showed that FBG was significantly higher in diabetic rats compared to control group (Table 3) and administration of extract of walnut leaves (150 and 250 mg/kg) in a dose‐dependent manner improved glucose in the treated groups, which is in agreement with other reports (Jelodar et al., 2007; Teimoori, Ghafarzadegan, & Hajiaghaee, 2010). Teimori et al. (2010) also reported an oral administration of 250 mg/kg BW per day of walnut leaves ethanol extract to alloxan‐induced diabetic rats (Teimoori et al., 2010). Moreover, Jelodar & Nazifi, (2001) demonstrated the anti‐diabetic effects of walnut powder mixed with food on diabetic rats. They attributed antidiabetic impacts of the walnut leaf to its insulin‐like substances. That is, serum glucose reduction after using the walnut leaf is completely justifiable due to the existence of such substances (Jelodar & Nazifi, 2001). Jelodar et al. (2007) reported treatment with walnut leaves reduces FBS and regenerate pancreatic beta cells in diabetic rats (Jelodar et al., 2007). The active ingredients of walnut leaves extract included quercetin, kaempferol, eugenol, avicularin, nicotine, caffeic acid, hyperin, beta‐eudesmol, juglone, p‐Coumaric acid, ascorbic acid, ellagic acid, gallic acid, neochlorogenic acid, and cyaniding (Delaviz et al., 2017). It can be said that anti‐hyperglycemic and hypoglycemic effects of walnut leaf extract are probably due to flavonoids, such as quercetin and kaempferol (Rabiei et al., 2018). Vessal, Hemmati, and Vasei (2003) reported that 10 and 15 mg/kg doses of quercetin had no impact on plasma glucose level in normal animals, but significantly decreased the level of plasma glucose in streptozotocin‐induced diabetic rats after 8–10 days, and blood glucose returned to the normal level (Vessal et al., 2003). It can be proposed that quercetin and kaempferol content of walnut leaf extract, act as an antioxidant and scavenger of free radicals, and have a role in beta cell regeneration and protects pancreatic islets. Their possible mechanisms can be associated with anti‐diabetic effect, anti‐oxidant effect (Alkhalidy et al., 2015; Kawser Hossain et al., 2016), impact on hepatic glucokinase (Vessal et al., 2003), inhibition of gastrointestinal absorption of glucose (Kwon et al., 2007), glucosuric effect (Beekmann et al., 2015), and/or insulin‐like external pancreatic mechanisms (Alkhalidy et al., 2015; Soares, Pereira Leal, Silva, Almeida, & Oliveira, 2017). In our study, concentrations of lipid profiles including TC, TG, and LDL‐c were significantly higher in diabetic rats compared to the control group. Different mechanisms and metabolic changes due to insulin deficiency are responsible for the increase in serum lipids (Lara‐Castro & Garvey, 2008). In the diabetic rats treated with walnut leaves extract levels of TC, HDL‐c, TG, VLDL‐c, and LDL‐c improved and did not show significant difference with the control group. It was previously observed that STZ‐ nicotinamide induced diabetic rats develop significant disturbances in lipid metabolism in the rat adipose tissue (Szkudelska, Nogowski, & Szkudelski, 2014). It is known that insulin exerts an anti‐lipolytic effect via phosphorylation of cGMP‐inhibited cAMP phosphodiesterase which leads to reduction in cAMP in the adipocytes (Czech, Tencerova, Pedersen, & Aouadi, 2013). It is rational that in the absence of insulin, the activity of hormone‐sensitive lipase adipocytes increases and causes disturbances in lipid metabolism. The liver plays an important role in the regulation of the metabolism of plasma lipoproteins. A decrease in lipid and lipoprotein production following improvement in glycemic condition and treatment with walnut leave extract noticed in diabetic rats. Bardini, Rotella, and Giannini (2012) showed a decrease in VLDL‐c production in diabetic patients following improvement of glycemic control (Bardini et al., 2012). The overall effect of walnut leave extract on VLDL‐c metabolism could be due to a decreas in VLDL‐c generation and increase of VLDL‐c removal. Hypocholesterolemic medicines reduce serum LDL‐c, possibly through stimulating receptor‐mediated remove of LDL‐c. This seems a possible explanation for walnut leave treatment, which shows a decrease in LDL‐c and no changes of HDL‐c in the control group.

An important role for oxidative stress and hyperhomocysteinemia in the development of type II diabetes mellitus has been proposed in the literature (Huang, Ren, Huang, & Li, 2013; Platt et al., 2017).

Although many studies have shown that there is a relationship between homocysteine and type II diabetes mellitus, a few studies reported that hyperhomocysteinemia is associated with type I diabetes mellitus or STZ‐induced diabetes. By following this evidence, the results of the present study show that plasma homocysteine level has a significant change in STZ‐induced diabetic rat. The positive association between insulin‐resistant and tHcy has been reported, indicating that the activity of enzymes involved in homocysteine metabolism may be affected by insulin levels (Masuda et al., 2008). It was reported that hyperinsulinemia is associated with an elevated homocysteine concentration and changes in key enzymes in homocysteine metabolism (Fonseca et al., 2000). It has been reported that insulin therapy in rats decreases the activity of cystathionine b‐synthase and increases methylenetetrahydrofolate reductase activity leading to elevated tHcy (Fonseca et al., 2000). As previously mentioned walnut leaves can recover pancreatic beta cells in diabetic rats and increase serum levels of insulin (Jelodar et al., 2007). Hence, it is likely that in this study decrease in homocysteine is due to the improvement of pancreatic beta cells and the increase in serum insulin level after treatment with the walnut leaves extract. These effects, which are probably due to the existence of quercetin and kaempferol active ingredients, are magnified by the increase in walnut leaf dose and experiment duration. Besides, during this study, mortality was not observed in diabetic or normal rats, receiving the walnut leaves extract.

## 5 CONCLUSION

The results of this study showed that cyclohexane extract of walnut leaves has an overall beneficial effect on body weight, fasting blood glucose, lipids profile, antioxidant enzymes activity, and homocysteine in an experimental model. This finding supports the traditional use of walnut leaves for controlling hyperglycemia in diabetics, hence further investigation with a longer period or the higher dose may show clearer features of this finding.

## CONFLICT OF INTEREST

The authors declare that they have no conflict of interest.
